# Use of mobile phones for rehabilitative services among prosthetics users in rural Acholi sub-region of northern Uganda: findings from a qualitative study

**DOI:** 10.1186/s12911-022-02008-z

**Published:** 2022-10-07

**Authors:** Walter Onen Yagos, Geoffrey Tabo Olok, Emmanuel Ben Moro, Jonathan Huck, Mahesh Nirmalan

**Affiliations:** 1 Department of Medical Library, Faculty of Medicine, Gulu University, Gulu City, Uganda; 2 Department of Computer Science, Faculty of Science, Gulu University, Gulu City, Uganda; 3 Department of Surgery, Faculty of Medicine, Gulu University, Gulu City, Uganda; 4 Department of Geography, School of Environment, Education and Development, University of Manchester, Manchester, UK; 5 Department of Social Responsibility and Public Engagement, Faculty of Biology, Medicine and Health, University of Manchester, Manchester, UK

**Keywords:** Ownership of mobile phone, Use of mobile phone applications, Prosthetics users, Prosthetics rehabilitative services

## Abstract

**Background:**

Digital technologies such as mobile phones have shown potential as vital tools for use in healthcare and related services. However, little has been done to explore its use for prosthetics rehabilitative services, especially in the Acholi sub-region of northern Uganda. We address this gap by exploring ownership of the mobile phone, knowledge of the use of mobile phone applications, use of mobile phones for prosthetics rehabilitative services and challenges faced in using the mobile phones.

**Methods:**

A case study design was used. We conducted semi-structured one-on-one interviews with 16 prosthetics users spread in the four districts of Nwoya, Amuru, Omoro, and Gulu of the Acholi sub-region of northern Uganda. We transcribed the data verbatim and explored the contents thematically to derive themes.

**Results:**

More prosthetics users (63%) owned mobile phones compared to those without (37%). Many who owned and use mobile phones are knowledgeable about applications for calls and messaging (47%). Some prosthetics users are knowledgeable in mobile money applications (21%), call applications only (16%) and, others were able to use the internet (16%). Many of the prosthetics users in this study use mobile phones to seek information, mainly relating to the management of prosthetics and treatment of diseases. Many participants were positive about the benefits of the use of mobile phones for prosthetics rehabilitation and related services. Common challenges affecting the use of mobile phones include the expensive price of airtime, few places for charging mobile phones, lack of electricity and inadequate skills to operate a mobile phone.

**Conclusion:**

The use of mobile phones can break down barriers created by distance and allow effective communication linkages between prosthetics users and rehabilitation services. Our results suggest that some prosthetics users owned mobile phones and used them to seek information relating to prosthetics rehabilitation services. We believe that promoting the use of the mobile phone for prosthetic rehabilitative services among prosthetics users is necessary and should be considered for practical and policy discussion relating to its use for prosthetics rehabilitation in rural areas.

## Background

Digital technologies such as mobile phones have shown a lot of potential for use in healthcare and related services [1, 2]. Healthcare workers and patients can use mobile phones for communicating a range of healthcare and related activities [3, 4] In Africa, studies have identified the use of mobile phones in various healthcare interventions. These include health consultations, education, data collection and reporting, patient monitoring and other healthcare-related communications [5]. Although much attention has been made to studying the use of mobile phones in healthcare [6], little has been done to examine its use for prosthetic rehabilitative services for lower-limb loss amputees [6]. Because of this, it is important to explore its use among prosthetic users in the Acholi sub-region of northern Uganda. In this study, we refer to the mobile phone as; a portable electronic communication device that enables one to make and receive calls, and send and receive text messages, with a camera and Internet functions.

Patients and healthcare workers can use mobile phones to facilitate the exchange of healthcare information between themselves [6]. A study by Ling et al. [6] suggests that interactions through mobile phones are becoming a common part of the health informatics ecosystem for rural areas. Patients and healthcare workers are continuing to use this technology to confront many healthcare-related challenges [1, 7]. These include among others health education, clinical and process improvements, management and treatment of diseases, and activity monitoring [1, 2, 6, 8].

The use of mobile phones for healthcare and related services is well documented [7]. However, little has been made to use qualitative research to investigate its use among amputees [6]. Qualitative research takes into account the perspective of the participant and can offer valuable insight into the problem, than what quantitative study could have provided. In Uganda and elsewhere, quantitative research has been used to target the general use of ICT among persons with disabilities [9, 10]. For instance, a study carried out in twelve regions of Uganda found disparities in the ownership of mobile phones among persons with disabilities. It showed high ownership among males (73%) than females (66%). In the Acholi sub-region, persons with disabilities were found to own fewer mobile phones (34%) compared to the other regions in Uganda [11]. Many of these studies overlooked what persons with disabilities used mobile phones for; especially the use of mobile phones concerning prosthetics rehabilitation services.

In Uganda, a lot of recommendations for improving access to prosthetics rehabilitation services have been made. These include using standard guidelines for treatment, expanding service hubs, and encouraging team-based care [12]. Despite these recommendations, the current service provision for persons with disabilities remains inadequate [13]. The use of mobile phones can provide opportunities in this area and support the current Government’s need for innovative solutions that break barriers to access to services for persons with disabilities in Uganda [14].

This particular group is economically disadvantaged and has a challenge in accessing rehabilitative services [14]. Their livelihoods are specifically affected by the cost of healthcare, transport, and low use of assistive products [14]. These situations are common in most parts of Uganda, especially in the Teso sub-region in Eastern Uganda and the Acholi sub-region of Northern Uganda [15].

The Acholi sub-region is among the regions in Uganda with the highest number of disabilities [16]. In addition, the latest statistics put the region among the poorest regions with poor health status [17]. Only 63.9% of the households in this region are within a 5-kilometre distance of public health facilities [16]. The region which measured about 29,174.19 square kilometres has only one government orthopaedic workshop situated at Gulu Regional Referral Hospital in Gulu City [13].

The challenges concerning access to healthcare and rehabilitative services by persons with disabilities are worrying. There is a need for solutions that can break barriers to access to healthcare and related services for persons with disabilities [14]. Fortunately, the use of mobile phones in other places has shown the possibilities in this area [2, 6–8]. However, little effort has been made to study its use in healthcare and rehabilitative services among amputees [6]. This study aims to explore the ownership of the mobile phone, knowledge of the use of mobile phone applications, use of mobile phones relating to prosthetics rehabilitative services and challenges prosthetics users face in using mobile phones.

## Methodology

### Study design and sites

The study used a case study design with multiple homogenous individuals [25]. Ancker et al. [26] argued that a case study is particularly valuable for evaluating interventions. We conducted this study in the four districts of Nwoya, Amuru, Omoro, and Gulu of the eight districts in the Acholi sub-region of northern Uganda. Participants were gathered at the health facilities of Alero HC III, Koch Goma HC IV, Langol HC II, Atiak HC IV, Lalogi HC IV, Lapinat HC III, Awach HC IV and Unyama HC III. These health facilities were used as hubs for interviews.

### Sampling

Participants in this study were sampled purposively based on their previous participation in the major limb loss (MLL) project that was supported by the Arts and Humanities Research Council (AHRC) in the United Kingdom and jointly implemented by Gulu University and Manchester University. In this project, 50 amputees spread across the 8 districts of the Acholi sub-region were fitted with new prosthetic devices between October and November 2019. We made prior telephone contacts with the coordinators to help mobilize the participants who were directed to the nearest health facility (hub). We called other participants directly to gather at the designated health facilities. This study targeted 20 prosthetics users from the districts of Nwoya, Amuru, Omoro, and Gulu. Sixteen (80%) reported to the designated hubs and were interviewed. We conducted the interviews on the 13th and 14th of December 2021. Each participant was given compensation of Uganda Shillings twenty thousand (about 6 USD) as a transport refund.

### Data collection

Semi-structured one-on-one interviews were conducted with 16 prosthetics users. Core guiding questions on the interview guide focused on; ownership of mobile phones, knowledge of using mobile phone applications, use of the mobile phone for prosthetics rehabilitative services, and challenges faced in using mobile phones. Interviews in each hub (health facility) were conducted in a separate room allocated by the in charge. Two of the investigators who are conversant with the Acholi, the dominant language spoken in the Acholi sub-region of northern Uganda conducted the interviews. These two investigators have received training and are experienced in qualitative research. One investigator asked questions and the other took notes during each interview. Each interview session lasted for about 25 and 40 min, approximately every interview took 32.5 min.

### Data analysis

Data from interviews were transcribed verbatim and the contents were thematically explored [18] to generate codes and themes for this study [19]. The raw interview data from the field were organized and typed in a table with 4 columns indicating; participant information, interview question, transcript and coding/theme. Transcripts were analysed manually and the following major themes and sub-themes emerged: they included ownership of the mobile phones (sub-theme: owned personal mobile phone, do not have a mobile phone, share a mobile phone, owned basic phones, and owned feature phones); knowledge of using mobile phone applications (sub-theme: knowledgeable in text messaging and calls, mobile money applications, calls applications only, able to use the internet); use of the mobile phone for prosthetics rehabilitative services (sub-theme: using mobile phones to seek for information, positive opinions about the benefits of the use of mobile phones, use of the mobile phone for remote health management); and challenges in using the mobile phone (sub-theme: cost of airtime, few places for charging, inadequate skills, poor network coverage). Two investigators knowledgeable in qualitative data analysis reviewed the transcripts independently to establish the authenticity of themes and findings. Themes were then presented and supported with accounts from participants, including direct quotes. Illustrative quotes from participants were represented with sex and age, for example; (Female age 45 years).

## Results

This study explored ownership of the mobile phone, knowledge of the use of mobile phone applications, use of mobile phones for prosthetics rehabilitative services and challenges faced in using the mobile phones. The analysis of the data obtained from interviews with prosthetics users was presented with the following major themes and sub-themes: ownership of the mobile phones (sub-theme: owned personal mobile phone, do not have a mobile phone, share a mobile phone, owned basic phones, and owned feature phones); knowledge of using mobile phone applications (sub-theme: knowledgeable in text messaging and calls, mobile money applications, calls applications only, able to use the internet); use of the mobile phone for prosthetics rehabilitative services (sub-theme: using mobile phones to seek for information, positive opinions about the benefits of the use of mobile phones, use of the mobile phone for remote health management); and challenges in using the mobile phone (sub-theme: cost of airtime, few places for charging, inadequate skills, poor network coverage).

### Participants

Sixteen prosthetics users participated in the one-on-one semi-structured interviews as categorized in Table 1. More male (75%) participants than females (25%) participated; the mean age was 47.3 years (range 23 to 75). More participants (63%) owned mobile phones than those without (37%). Most of the participants were peasant farmers (81%), farming mainly for subsistence use. Generally, most households in the Acholi sub-region of northern Uganda are in a subsistence economy, characteristically poor and practiced subsistence agriculture [17].

**Table 1:** Participant demographics of (*n* = 16)

Gender		Frequency	Percentage
	Female	4	25
	Male	12	75
Ownership of phone			
	Yes	10	63
	No	6	37
Age range			
	23–33 Years	1	6
	31–41 Years	5	31
	42–52 Years	4	25
	53–63 Years	5	31
	64–74 Years	0	0
	75 Above Years	1	6
Mean age	*47.3 (23 to 75)*		
Occupation			
	Peasant farmer	13	81
	Small business	1	6
	Civil servant	1	6
	Student	1	6

### Ownership of mobile phone

The interviews demonstrated that ten (63%) out of sixteen prosthetics users owned mobile phones; the remaining six (37%) do not own a mobile phone. Of the six prosthetics users who do not own mobile phones, four (67%) do not have them at all, while two (33%) share mobile phones with spouses or children when needed. During the interviews, it was observed that, out of the ten prosthetics users who owned mobile phones, six (60%) owned basic mobile phones, while four (40%) owned feature phones. The basic mobile phone is limited to voice calls and text messages, while the feature phone has limited applications and browsing capability and functionality. Although this study is distinctive to prosthetics users, available evidence showed the percentage of ownership of mobile phones from all kinds of persons with disabilities in the Acholi sub-region at only 34% [20].

### Knowledge of using mobile phone applications

Although prosthetics users are knowledgeable in mobile phone applications, the interviews indicated that there are disparities in sentiments among prosthetics users about the knowledge they have about using mobile phone applications. Of the sixteen participants in this study, twelve (75%) used mobile phones. Responses showed that most of the participants are knowledgeable in using mobile phone text messaging and call applications (47%). Participants consider the knowledge of using mobile phone applications as being able to use mobile phone applications for calls, text messages, internet and mobile money. The quotes bellow illustrates their opinions:

“I am knowledgeable about making and receiving calls, and can send and receive messages using my mobile phone (Male, age 38 years)”.

Some prosthetics users are knowledgeable in mobile money applications (21%); some are knowledgeable about call applications only (16%) and, others, were able to use the internet for accessing e-mail and social media (16%). Generally, the use of the internet among persons with disabilities in Uganda is low [10, 17]. The quotes underneath show knowledge of some of the combinations of mobile phone applications:

“I can make and receive calls, send text and receive messages, and able to use the internet and mobile money applications as well (Male, age 23 years)”.

“As a coordinator…I know how to use a mobile phone; I can call, send text messages and also use mobile money applications (Male, age 40 years)”.

### Use of mobile phone for prosthetics rehabilitative services

The interviews demonstrated that prosthetics users are in positions to use mobile phones for various activities relating to prosthetics rehabilitation services. Many of the prosthetics users in this study use mobile phones to seek information about new materials and available rehabilitative services offered by the Orthopaedic Worksop at Gulu Regional Referral Hospital. This is illustrated by the following quotes:

“I normally call the orthopaedic workshop at Gulu Regional Referral Hospital in case I have problems with the prosthetics or the stump…one time I called the workshop to find out if they have brought new materials so that I go and change my old already damaged artificial limb…the doctors from orthopaedic workshop responded very well and gave me the information which I require (Male, age 47 years)”.

“I used a mobile phone to contact the workshop mainly on the problem associated with the limb…mobile phone is useful for me to connect to the hospital sometime to understand if new materials have arrived (Male, age 38 years)”.

Further, prosthetics users used mobile phones to facilitate remote management of prosthetics and treatment of diseases. Participants continuously seek information that can help them in the management of their conditions, such as diseases, use of artificial limbs and treatment of wounds among others.

“I use my phone to connect to the orthopaedic workshop very frequently mainly about my limb, the level of sugar….you know I am diabetic so I use the phone to consult the doctors on other health issues so that they can advise me on food and other things that I should not do (Male, age 47 years)”.

“I normally find a problem when I am walking and climbing walkway...steps in school. I called the workshop people and I was advised to use the stronger leg first when climbing or coming down the steps (Male, age 23 years).”

“I use the phone to call the hospital if I am lacking anything related to my condition, for example, I call the hospital to get help on how to manage and treat the wound on my stump (Male, age 42 years)”.

The interviews also demonstrated that prosthetics users had positive opinions about the benefits of using mobile phones for prosthetics rehabilitation and related services. The use of mobile phones by participants seems to aid mobilization and coordination, break barriers of distance and allow easy access to information. Participants narrated some of the following, emphasizing the benefits of mobile phones:

“I am one of the coordinators, but also a prosthetics user. I always connect with the people of the workshop on issues concerning limbs and problems with stumps using my mobile phone. My work as a coordinator is complex because I receive phone calls from many people. I get phone calls from the orthopaedic workshop, from the prosthetics users, and even from new amputees who require artificial limbs. I hope you have seen the old man which I came with…he was amputated and needs a limb (Male, age 40 years)”.

“I have no mobile phone and I have never used it to call the coordinator. I normally walk physically to the coordinator’s home which is about 1 kilometre to get information. If I had a mobile phone, it would have been better (Male, age 54 years)”.

“I do not have a mobile phone, but I can see the benefits from other people, you can get information easily; can connect to doctors on issues pertaining amputees and many other things (Male, age 55 years)”.

### Challenges faced by prosthetics users in using mobile phone

Although prosthetics users appear to use mobile phones for healthcare-related issues, many challenges might hinder the use of mobile phones in the rural Acholi sub-region. The challenges include: participants could not afford airtime because of the expensive price; the few places for charging phones are associated with a lack of electricity and solar among participants, inadequate skills mean lack or inadequate skills to operate mobile phones, and poor network coverage, is associated with the weak phone networks coverage in the rural areas. The following quotes from participants show some of the challenges:

“The problem I normally encounter is the issue of the cost of buying airtime and charging my phone (Male, age 59 years)”.

“I told you before that I don’t know how to use it but I need to learn how to use the mobile phone (Male, age 38 years)”.

“I have no phone, but can talk with it when my son gives me. My son helps me to communicate with the phone. I particularly do not know how to use the mobile phone (Female, age 59 years)”.

“What I know is that many of the prosthetics users in my area wait for other people to call them. They rarely make calls to other people or me the coordinator and the major reason for this is the cost of airtime. People find it difficult to sacrifice money for airtime in place of other necessities such as food and treatment. But to avoid this problem of airtime, the organizations that help us normally give airtime to coordinators to help in mobilization…but network in our area is also very weak (Male, age 40 years)”.

## Discussion

Over the years, the use of mobile phones in healthcare and related services has shown tremendous benefits. This is seen in the areas of health education, treatment and healthcare management [1, 2, 6, 8]. This study explores the ownership of the mobile phone, knowledge of the use of mobile phone applications, use of mobile phones relating to prosthetics rehabilitative services and challenges prosthetics users face in using a mobile phone.

The findings of this study showed that more prosthetics users interviewed owned mobile phones compared to those without. However, the lack of ownership of a mobile phone for the few prosthetics users does not always translate to lack of use. Further, through close observations and interactions, it was revealed that participants mostly owned a basic mobile phone. This is similar to previous findings where persons with disabilities in this region reported owning mostly basic mobile phones [5, 11]. It is therefore most likely that participants in this study can use only basic mobile phone applications. It also emerged that many prosthetics users who owned and use a mobile phones are knowledgeable about calls and messaging applications. Very few prosthetics users had some knowledge of the internet and use the applications for e-mail and social media purposes. The reasons why participants’ knowledge is restricted to making calls and messaging could be associated with the types of mobile phones and skills. It was observed that the few who use the internet applications possessed feature phones, probably a reflection of the extension in the use of applications. The feature phone has limited applications and browsing capability and functionality. Our study is unique in the sense that it targets only prosthetics users with lower-limb loss. Previous studies had little consideration in segregating disability groups while studying the use of mobile phones [9–11]. Notwithstanding, our findings may be relevant for future interventions that could leverage interest in the use of the mobile phone to communicate healthcare information relating to prosthetics rehabilitation in rural areas.

Further, several prosthetics users who owned mobile phones held opinions that they use the mobile phone to communicate with the orthopaedic workshop at Gulu Regional Referral Hospital. It became apparent from the participants that they call the workshop to seek prosthetics rehabilitative services. They called to seek information about new prostheses, diseases, managing wounds and training on the use of prostheses. The majority of the prosthetics users in the Acholi sub-region of northern Uganda are stationed in rural and remote areas, and they lack access to healthcare and related services [13, 14]. The use of the mobile phone by participants in this study therefore presents a huge opportunity. Previous studies have reported the use of the mobile phone in health [1, 7], and a lot more have shown its benefits in the areas of health education, treatment and healthcare management [1, 2, 6, 8]. Scaling up the use of mobile phones to target groups such as prosthetics users can break barriers to access to healthcare services [1, 7].

Despite the prospective potential for future use of the mobile phone for prosthetics rehabilitative services, important challenges remain. Many prosthetics users held concerns that they faced challenges as a result of the high cost of airtime, few places for charging phones and insufficient skills they have in using a mobile phone. These challenges are common phenomena in rural areas in Uganda and other places [10, 11]. Some of these challenges could have arisen due to other bigger problems. The Acholi sub-region of northern Uganda has the highest number of poor households, without electricity compared to other regions in Uganda [11, 17]. Promoting the use of the mobile phone for prosthetic rehabilitative services among prosthetics users is necessary, but with consideration of the factors that hinder the use of mobile phones. We believe that extensive use of the mobile phone can break down barriers associated with distance and create effective communication linkages between prosthetics users and rehabilitation services.

### Study limitations

We purposively targeted a few prosthetics users who were previously under the major limb loss project for interviews. Given our rather small sample size (*n*-16), we were only able to offer limited insights regarding ownership of the mobile phone, knowledge of the use of mobile phone applications, use of mobile phones relating to prosthetics rehabilitative services and challenges that prosthetics users faced in using the mobile phone. One of our limitations is that we may have not adequately addressed all issues relating to the use of mobile phones. These include areas such as cost, types of phones, skills and disproportions of use by gender among others. We believe that future studies should address these areas, especially targeting prosthetics users and other groups of persons with disabilities in rural and urban areas. Much as we targeted a homogenous group with a similar disability, the small sample size has limited the study in terms of population coverage. We would recommend a mixed-methods study to investigate the use of the mobile phone for healthcare and rehabilitative services while considering other stakeholders such as healthcare workers.

Notwithstanding, we believe that this study is the first of its kind to target the use of mobile phones among lower limb amputees who are prosthetics users in rural areas in Uganda. We further believe that participants ‘revelations in this study were grounded on their own experiences and opinions regarding ownership of the mobile phone, knowledge of the use of mobile phone applications, use of mobile phones relating to prosthetics rehabilitative services and challenges that they faced in using the mobile phone.

## Conclusion

The use of mobile phones can break down barriers created by distance and allow effective communication linkages between prosthetics users and rehabilitation services. We explored ownership of the mobile phone, knowledge of the use of mobile phone applications, use of mobile phones relating to prosthetics rehabilitative services and challenges that prosthetics users faced in using mobile phones in the rural Acholi sub-region of northern Uganda. Despite the challenges in using a mobile phone, our findings suggest that some prosthetics users owned a mobile phone and used it for communicating issues relating to prosthetics rehabilitations. With consideration of the factors that hinder the use of mobile phones, we believe that promoting the use of mobile phones for prosthetic rehabilitative services among prosthetics users is necessary and should be considered for practical and policy discussion relating to its use for prosthetics rehabilitation in rural areas.

## Data Availability

Data supporting the conclusions of this article are all included in the paper.

## References

[1] Krishna S, Boren SA, Balas EA (2009). Healthcare via cell phones: a systematic review. Telemedicine and e-Health.

[2] Onweni CL, Venegas-Borsellino CP, Treece J, Turnbull MT, Ritchie C, Freeman WD. The Power of Mobile Health: The Girl with the Gadgets in Uganda. Mayo Clinic Proceedings: Innovations, Quality & Outcomes. 2021 Apr 1; 5(2):486 – 94. 10.1016/j.mayocpiqo.2021.01.001 PMC8105515 33997644

[3] Binedell T, Subburaj K, Wong Y, Blessing LT. Leveraging digital technology to overcome barriers in the prosthetic and orthotic industry: Evaluation of its applicability and use during the COVID-19 pandemic. JMIR Rehabilitation and Assistive Technologies. 2020 Nov 5; 7(2):e23827. 10.2196/23827 PMC7677018 33006946

[4] LeFevre AE, Shah N, Bashingwa JJ, George AS, Mohan D. Does women’s mobile phone ownership matter for health? Evidence from 15 countries. BMJ global health. 2020 May 1; 5(5):e002524. 10.1136/bmjgh-2020-002524 PMC7245424 32424014

[5] Albabtain AF, AlMulhim DA, Yunus F, Househ MS. The role of mobile health in the developing world: a review of current knowledge and future trends. Cyber Journals: Multidisciplinary Journals in Science and Technology [JSHI]. Journal of Selected Areas in Health Informatics. 2014:10–5.

[6] Ling R, Poorisat T, Chib A. Mobile phones and patient referral in Thai rural healthcare: A structuration view. Information, Communication & Society. 2020 Feb 23; 23(3):358 – 73.

[7] Debeljak M, Vidmar G, Matjacic Z, Burger H (2020). Information and communication technology used by people with lower limb loss in Slovenia. Int J Rehabilitation Res Int Z Fur Rehabilitationsforschung Revue Int De Recherches De Readaptation.

[8] Uganda Communications Commission (2021). Market performance report Quota 21.

[9] Amarakoon P, Hewapathirana R, Dissanayake VH. MHealth in the public health sector: challenges and opportunities in low-and middle-income countries: a case study of Sri Lanka. In *Digital Health. 2021* (pp. 123–142). Elsevier.

[10] Feroz A, Jabeen R, Saleem S (2020). Using mobile phones to improve community health workers performance in low-and-middle-income countries. BMC Public Health.

[11] Chadwell A, Diment L, Micó-Amigo M, Morgado Ramírez DZ, Dickinson A, Granat M, Kenney L, Kheng S, Sobuh M, Ssekitoleko R, Worsley P (2020). Technology for monitoring everyday prosthesis use: A systematic review. J Neuroeng Rehabil.

[12] Uganda Bureau of Statistics. The National Population and Housing Census 2014 – National Analytical Report on persons with disabilities, Kampala, Uganda. 2019.

[13] Uganda Communications Commission (2018). Access and Usage of Information and Communications Technologies (ICTS) by People with Disabilities (PWDS) in Uganda, draft report.

[14] Aranda-Jan C (2020). The Mobile Disability Gap Report 2020.

[15] Unit A. Access to Health Care Services. Experiences of Persons Living with Disabilities in Eastern and Northern Uganda. 2019.

[16] Arifin N, Hasbollah HR, Hanafi MH, Ibrahim AH, Rahman WAWA, Aziz RC (2017). Provision of prosthetic services following lower limb amputation in Malaysia. Malaysian J Med sciences: MJMS.

[17] Ministry of Gender. Labour and Social Development. Harnessing Their Potential, the State Of Disability in Uganda, Summary Report. Kampala, Uganda. 2020.

[18] Okello TR, Magada SM, Atim P, Ezati D, Campion A, Moro EB, Huck J, Byrne G, Redmond A, Nirmalan M. Major limb loss (MLL): an overview of aetiology, outcomes, experiences and challenges faced by amputees and service providers in the post-conflict period in Northern Uganda. Journal of global health. 2019 (3): 1–14.

[19] Uganda Bureau of Statistics. The National Population and Housing Census 2014 – Health status and associated factors. Thematic Report Series, Kampala, Uganda. 2017.

[20] Uganda Bureau of Statistics (2021). The Uganda national household survey 2019/2020.

